# Repeated inhibition of sigma-1 receptor suppresses GABA_A_ receptor expression and long-term depression in the nucleus accumbens leading to depressive-like behaviors

**DOI:** 10.3389/fnmol.2022.959224

**Published:** 2022-09-30

**Authors:** Yaoyao Qin, Weixing Xu, Kunpeng Li, Qi Luo, Xi Chen, Yue Wang, Lei Chen, Sha Sha

**Affiliations:** Department of Physiology, Nanjing Medical University, Nanjing, China

**Keywords:** sigma-1 receptor (σ_1_R), nucleus accumbens (NAc), GABA_A_ receptor (GABA_A_R), depressive-like behaviors, long-term depression (LTD)

## Abstract

Sigma-1 receptor (σ_1_R) downregulation in male mice is known to cause a depressive-like phenotype. The nucleus accumbens (NAc), a region associated with affective regulation, has high levels of σ_1_R. Here, we investigated the effect of repeated inhibition of σ_1_R in the NAc on depressive-like behaviors and synaptic plasticity by microinjecting σ_1_R antagonist NE-100 into NAc nuclei in mice (NE-100 mice); this was followed by behavioral tests and field potentials recordings. We first examined the effect of NE-100 administration on σ_1_R expression and found that cell surface levels of σ_1_R were significantly reduced in the NAc of NE-100 mice. Compared to control mice, NE-100 mice exhibited significantly prolonged immobility in forced swim test (FST) and tail suspension test (TST), impaired long-term depression (LTD) as well as multi-spike waveform field excitatory postsynaptic potential (fEPSP) with an extended duration and an increased paired-pulse ratio (PPR). Reduced levels of GABA_A_ receptor (GABA_A_R)-α1, -α2, -β2, and -β3 subunits, membrane D2R, and PKC phosphorylation in the NAc were observed in NE-100 mice. Activation of GABA_A_R by muscimol corrected the extended fEPSP duration and increased PPR, restored LTD maintenance as well as alleviated depressive-like behaviors in NE-100 mice. The decline of PKC phosphorylation in the NAc of NE-100 mice was corrected by injecting NAc with quinpirole, a D2R agonist. Injections of quinpirole or PMA (a PKC activator) into NAc of NE-100 mice rescued the expression levels of GABA_A_R, and alleviated the increase in PPR and impairment in LTD; these effects were sensitive to GF109203X, a PKC inhibitor. Furthermore, injecting NAc with quinpirole or PMA relieved depressive-like behaviors in NE-100 mice. Collectively, these results indicate that repeated inhibition of σ_1_R in the NAc reduces D2R-mediated PKC phosphorylation and suppresses GABA_A_R expression, thus impairing LTD maintenance and leading to depressive-like behaviors.

## Introduction

Sigma-1 receptors (σ_1_Rs) are highly expressed in regions of the brain involved in emotion and neuropsychiatric disorders (Maurice et al., 2002; Lan et al., 2019). Animal models and clinical trials have confirmed that σ_1_R agonists exert antidepressant effects (Hayashi et al., 2011) and that mice with σ_1_R deficiency exhibit a depressive-like phenotype (Sabino et al., 2009; Sha et al., 2015; Zhang S. et al., 2017). However, the mechanisms underlying these effects have yet to be fully elucidated.

Several lines of investigation indicate that σ_1_Rs are involved in the activities of multiple neurotransmitter systems in the brain. Agonists of σ_1_R can influence intracellular Ca^2+^ levels by regulating Gq-coupled receptors and by mediating the steady-state balance of dopaminergic neurons (Hayashi and Su, 2007; Ryskamp et al., 2017). Interactions of σ_1_R and dopamine receptors or dopamine receptor-containing heteromers can indirectly regulate food-seeking behavior (Aguinaga et al., 2019). Brünig et al. (1999) reported that D2 receptor (D2R) activation results in an enhancement of GABAergic transmission involving the protein kinase C (PKC) pathway in the olfactory bulb. GABAergic neurons have been found to play a major role in controlling mood and stress. Deficits in the functionality of the cortical GABAergic system resulting from exposure to chronic stress can compromise the integrity of neurocircuits and lead to depression and other stress-related disorders (Fogaça and Duman, 2019). The σ_1_R has been reported to modulate GABA release, GABA transport at the presynaptic level, and the activity of the GABA type A receptor (GABA_A_R; Mtchedlishvili and Kapur, 2003; Pozdnyakova et al., 2020).

A significant level of σ_1_R has been reported in the nucleus accumbens (NAc; Hayashi et al., 2010; Delint-Ramirez et al., 2020), a region that is critical for reward and motivation. Alterations in the NAc have been implicated in the pathophysiology of depression (Bagot et al., 2015). GABAergic medium spiny neurons (MSNs), which co-express D1 and D2 receptors (D1R and D2R), are the major cell type in the NAc and receive glutamatergic inputs from the ventral hippocampus (vHIP) and basolateral amygdala (BLA) amongst many other inputs (Nicola et al., 2000). Projections from the vHIP-NAc and BLA-NAc have been shown to regulate emotional behavior, social interaction behavior, and sensitivity to depression in mice (Sesack and Grace, 2010; Bagot et al., 2015; Muir et al., 2020). Exposure to stress has been shown to impair long-term depression (LTD) in the NAc and induces depressive-like behaviors in mice (Wang et al., 2010). Our previous studies confirmed that in the BLA of mice, a reduction of dopaminergic function suppresses GABA_A_R (Zhang T. et al., 2017). Furthermore, σ_1_R knockout weakens the GABA_A_R-mediated inhibition and leads to impaired synaptic plasticity and depressive-like behaviors (Zhang B. et al., 2017). Therefore, it is of great interest to investigate whether a reduction of σ_1_R in the NAc would affect the functionality of local GABAergic and dopaminergic neurons, and thus impact depressive-like behaviors. Our previous study demonstrated that the intracerebroventricular injection of NE-100 for 3 days in wild-type mice results in the same biological changes as in the σ_1_R knockout mice (Di et al., 2017). It has been shown that activated σ_1_R dissociates from the chaperone-binding immunoglobulin protein and is transferred from the mitochondria-associated endoplasmic reticulum membrane to other sites, such as the cell surface, or cytoplasm. NE-100 as an antagonist of σ_1_R can prevent this process (Hayashi and Su, 2007). In the present study, we used σ_1_R antagonists to microinject NAc nuclei in male ICR mice and assayed σ_1_R expression to clarify its activity; then investigated the influence of NAc-injection with NE-100 in the NAc on depressive-like behaviors and synaptic plasticity. To investigate the mechanisms underlying these effects, we also investigated the expression levels of GABA_A_R and dopamine receptors as well as the phosphorylation of PKC in the NAc. Finally, we analyzed the causal link between synaptic plasticity and the depressive-like phenotype in mice receiving repeated inhibition of σ_1_R. Collectively, our results indicate that repeated inhibition of σ_1_R in the NAc reduces the expression levels of GABA_A_R and impairs the maintenance of LTD, thus causing a depressive-like phenotype.

## Material and Methods

### Animals

All animal experiments were performed in accordance with the ARRIVE guidelines of Laboratory Animal Care (Kilkenny et al., 2012). All mice were handled in accordance with the experimental animal guidelines of the Laboratory Animal Research Institute and Ethical Committee of Nanjing Medical University (No. 2016-110). All efforts were made to minimize animal suffering and to reduce the number of animals used. For instance, laboratory personnel handled these mice carefully, used appropriate anesthesia during surgery, and kept the mice warm on a constant temperature blanket following surgery. Twelve-week-old male ICR mice (Animal Core facility of Nanjing Medical University) were maintained under constant environmental conditions (temperature 23 ± 2°C, humidity 55% ± 5%, and a 12:12 h light/dark cycle) with free access to water and food. The study had not been pre-registered with a pre-specified endpoint. This study was exploratory and there were no pre-determined exclusion criteria for the animals.

### Reagents and antibodies

σ_1_R antagonist NE-100 (Tocris Cat#3133), PKC activator phorbol 12-myristate 13-acetate (PMA; Sigma-Aldrich Cat#P1585), PKC inhibitor GF109302X (Tocris Cat#0741), D2R antagonist L-sulpiride (Sigma-Aldrich Cat#S7771), D2R agonist quinpirole (Sigma-Aldrich Cat#Q102), GABA_A_R agonist muscimol (Alomone Labs Cat#M-240), GABA_A_R antagonist bicuculline(Sigma-Aldrich Cat#14340), D1R agonist SKF38393 (Sigma-Aldrich Cat#S101), D1R antagonist SCH23390 (TocrisCat#No. 0925), NMDA receptor (NMDAR) agonist NMDA (Sigma-Aldrich Cat#M3262) and CB1 receptor agonist WIN55, 212-2 (MedChemExpress Cat#HY-13291) were dissolved in dimethyl sulfoxide (DMSO) and diluted by sterile saline or artificial cerebrospinal fluid (ACSF; in mM: 124 NaCl, 2 CaCl_2_, 4.5 KCl, 1.0 MgCl_2_, 1.2 NaH_2_PO_4_, 10 D-glucose, and 26 NaHCO_3_, pH7.4) to a final concentration of 0.1% DMSO. We used triple antibiotic ointment containing 400 units of bacitracin Zinc, 5 mg of neomycin dulfate, and 5,000 units of polymyxin B sulfate (WATER-JEL Technologies Cat#22405). Several antibodies were used in this study: anti-D2R (1:1,000; Millipore Cat#AB5084p, RRID:AB_2094980), anti-GABA_A_R-α1 (Sigma Cat#G4416, RRID:AB_477016), anti-GABA_A_R-α2(AbcamCat#ab72445, RRID:AB_1268929), anti-GABA_A_R-β2 (Affinity Biosciences Cat#DF6671, RRID:AB_2838633), anti-GABA_A_R-β3 (Abcam Cat#ab98968, RRID:AB_10670809), anti-phosphorylation PKC (Epsilon Ser729; Abcam Cat#ab63387, RRID:AB_1142277), anti-PKC (Abcam Cat#ab253274, RRID:AB_2827663), anti-σ_1_R (B5; Santa Cruz Biotechnology Cat#sc-137075, RRID:AB_2285870), anti-Na^+^-K^+^ ATPase (Cell Signaling Technology Cat#3010, RRID:AB_2060983) and anti-GAPDH (Abcam Cat#ab181602, RRID:AB_2630358).

### Drug administration

For NAc micro-injections, mice were anesthetized with an injection (i.p) of ketamine (100 mg/kg)/xylazine (10 mg/kg; Kroeger et al., 2017) in accordance with institutional guidelines and then placed in a stereotaxic instrument (Stoelting, Wood Dale, IL, USA). A 26-gauge stainless-steel guide cannula (Plastics One, Roanoke, VA, USA) was implanted into the unilateral NAc shell (anterior to the bregma: +1.54 mm; middle lateral: ±0.7 mm; dorsal ventral: −4.5 mm; Zhang et al., 2021). On the third postoperative day, the dummy cannula was removed from the guide cannula and then replaced by an infusion cannula (30 gauge) connected by polyethylene tubing (PE10; Becton Dickinson, Sparks, MD, USA) with a stepper motorized micro-syringe (Stoelting). NE-100 (0.15 nmol/mouse), L-sulpiride (0.25 μg/mouse), quinpirole (0.5 μg/mouse), PMA (48 pmol/mouse), GF109203X (5 ng/mouse), muscimol (4 nmol/mouse), NMDA (5 nmol/mouse) or WIN55, 212-2 (1.5 μg/mouse) was injected in a volume of 0.25 μl/side NAc (Yang et al., 2011; Madronal et al., 2012; Zhang T. et al., 2017; Zhang B. et al., 2017). Control mice were given an equal volume of vehicle. Only the administered NAc regions were used for mRNA and protein tests, as well as electrophysiological experiments. The micro-injection sites were validated postmortem by an injection with 2% Evans Blue followed by histological detection. After surgery, a triple antibiotic ointment was applied copiously on the closure site for three consecutive days.

For bath applications involving brain slices, the mice were decapitated under deep anesthesia with isoflurane (5%; Di et al., 2020). Then, the brains were rapidly removed and coronal brain slices containing the NAc region (350 μm) were cut using a vibrating microtome (Microslicer DTK 1500, Dousaka EM Co, Kyoto, Japan) in an ice-cold oxygenated (95% O_2_/5% CO_2_) cutting solution composed of (in mM): 94 sucrose, 30 NaCl, 4.5 KCl, 1.0 MgCl_2_, 26 NaHCO_3_, 1.2 NaH_2_PO_4_, and 10 D-glucose (pH 7.4). Our previous studies reported that the bath-application of muscimol (10 μM) and bicuculline (10 μM) to brain slices for 30 min could alter synaptic transmission in field potential recordings (Zhang T. et al., 2017; Di et al., 2020). Thus, in this study, the brain slices were treated with muscimol (10 μM) or bicuculline (10 μM) for 30 min. In all pharmacological experiments, the control slices were treated with an equal volume of vehicle.

### Experimental design and groups

In total, we used 260 mice: control mice (*n* = 120) and NE-100 mice (*n* = 140). The mice were divided into three experimental groups. The first group (12 + 12 control mice and 12 + 12 NE-100 mice) was used to examine the influence of inhibiting σ_1_R in the NAc on depressive-like behaviors, the electrophysiological characteristics of the NAc, and the expression levels of GABA_A_R and the dopamine receptor. At the end of the behavioral tests, the mice were divided into two cohorts, one cohort for field potential recording and the other for the detection of mRNA and protein. The second group (48 control mice and 48 + 20 NE-100 mice) was used to test whether reduced levels of σ_1_R in the NAc altered the expression levels of GABA_A_R *via* D2R-mediated PKC activity; this group was also used to examine the effect of suppressed D2R/PKC/GABA_A_R levels on LTD. The third group (48 control mice and 48 NE-100 mice) was used to investigate the effect of suppressed D2R/PKC/GABA_A_R levels on mouse affective disorder. The second and the third groups of control mice received either non-coadministered quinpirole, L-sulpiride, GF109203X, PMA, or muscimol; NE-100 mice received non-coadministered uinpirole, L-sulpiride, PMA, or muscimol, orcoadministered quinpirole and GF109203X. The volume of the microinjected drug was limited to 0.25 μl.

### Field potential recording

Brain slices were transferred to a recording chamber and continuously perfused with oxygenated ACSF and maintained at 30 ± 1°C. Field potential recordings were performed in the NAc, the area immediately surrounding the anterior commissure. Field excitatory postsynaptic potentials (fEPSPs) were evoked by a concentric bipolar stimulation electrode (FHC, St Bowdoin, ME, USA) that was placed 300–500 μm away from the recording electrode (Wang et al., 2010; White et al., 2016). Constant current pulses (0.1 ms, 0.05 Hz) were supplied by a stimulator (SEN-3301, Nihon Kohden, Japan). To record the fEPSPs, glass pipettes (4–5 MΩ) were filled with 2 M NaCl and connected to a neutralized, high input-impedance preamplifier with a high-pass filter at 5 kHz. Signals were amplified by a differential AC amplifier (A-M Systems, model 1700, Seattle, WA, USA) and were digitized and saved using the pCLAMP system (Axon Instrument Inc., Sunnyvale, CA, USA). A stable baseline of fEPSPs was recorded for at least 20 min by using a stimulus intensity adjusted to elicit approximately 50% of its maximal amplitude, before applying drugs or delivering low-frequency stimulation (LFS; 10 Hz, 10 min; Wang et al., 2010). Baseline synaptic transmission was assessed by averaging the response to six pulses (from 0.1 to 1.1 mA). The fEPSP slope was calculated as the absolute value of the rising phase between 10% and 90% of the first negative peak response, by using Clampfit 10.0 (Molecular Devices; White et al., 2016). The paired-pulse ratio (PPR) was measured by using the intensity of the test stimulus with an inter-pulse interval (IPI) of 15–100 ms. For LTD evaluation, pre-train responses were recorded for 20 min (baseline); this was followed by LFS. Single-pulse recording resumed immediately after the LFS-train had been delivered and continued for 60 min.

### Reverse transcription polymerase chain reaction (RT-PCR)

Mice were anesthetized with ketamine (100 mg/kg)/xylazine (10 mg/kg) and the brains were quickly removed. Then, coronal sections (500 μm in thickness) from +1.5 mm to +0.5 mm relative to the bregma were cut using a cryostat microtome (CM1900, Wetzlar, Hessen, Germany) according to the Mouse Brain Atlas (Paxinos and Franklin, 2001). The region containing the NAc was then harvested using a 15-gauge needle (inner diameter: 1.5 mm) and RNA was extracted using TRIzol reagent kit (Invitrogen, Camarillo, CA, USA). Total RNA was then reverse transcribed into cDNA using a Prim Script RT reagent kit (TaKaRa, China) for quantitative PCR (ABI Step One Plus, Foster City, CA, United States) in the presence of a fluorescent dye (SYBR Green I; Takara, China). The primer sets used for *σ_1_R*, *GABA_A_R-α1*, *GABA_A_R-α2*, *GABA_A_R-α3*, *GABA_A_R-α4*, *GABA_A_R-α5*, *GABA_A_R-β1*, *GABA_A_R-β2*, *GABA_A_R-β3*, *GABA_A_R-γ1*, *GABA_A_R-γ2*, *GABA_A_R-γ3*, *GABA_A_R-δ*, *D1R*, and *D2R* were designed according to previous publications (Nakai et al., 2014; Pan et al., 2017; Chen et al., 2018). The relative expression of genes was determined using the 2^−ΔΔCt^ method and normalized to *GAPDH* expression. Final values were averaged from four sets of independent experiments and were expressed as a percentage of control mice.

### Cell-surface biotinylation

NAc slices were placed on a 6-well plate and washed with frozen ACSF for 5 min. Then, the slices were incubated with ACSF containing EZ-link Sulfo-NHS-SS-Biotin (0.5 mg/ml, Pierce, Northumberland, UK) for 25 min at 4°C. Next, the slices were washed three times with ACSF containing 50 mM NH_4_Cl (5 min per wash) at 4°C to remove excess biotin. After biotinylation, the NAc region was removed by dissection and homogenized with lysis buffer containing 50 mM Tris-HCl (pH 7.4), 150 mM NaCl, 1.5 mM MgCl_2_, 1 mM EGTA, 0.5 mM DTT, 50 mM NaF, 2 mM sodium pyruvate, 25% glycerol, 1% Triton X-100, 0.5% sodium deoxycholate, and 1% protease inhibitor cocktail. After centrifugation at 20,000× *g* for 20 min at 4°C, the supernatants were collected as the source of protein and the final protein concentration was determined using a bicinchoninic acid (BCA) protein assay kit (Pierce Biotechnology, Rockford, IL, USA). Biotinylated proteins (50 μg) were then incubated with streptavidin-coated magnetic beads (30 μl) on a head-over-head shaker for 45 min at room temperature. The streptavidin beads to which the biotinylated proteins had adhered were washed three times with lysis buffer containing 0.1% sodium dodecyl sulfate (SDS) and then separated with a magnet. The biotinylated proteins were eluted into sample buffer (62.5 mM Tris-HCl, 2% SDS, 5% glycerol, 5% 2-mercaptoethanol) at 100°C for 5 min. The protein lysates and biotinylated proteins (cell surface) were then frozen until analysis.

### Western blotting analysis

Proteins from the NAc region were separated by 10% SDS-polyacrylamide gel electrophoresis and transferred onto a polyvinylidene difluoride membrane (Millipore, MA, USA). Non-specific binding sites were blocked with 5% non-fat milk in tris-buffered saline containing 0.1% Tween-20 (TBST) for 1 h. Then, the membranes were incubated at 4°C overnight with a monoclonal antibody to D2R (1:1,000), antibodies to GABA_A_R-α1 (1:1,000), GABA_A_R-α2 (1:1,000), GABA_A_R-β2 (1:1,000), GABA_A_R-β3 (1:1,000), an antibody to phosphorylated PKC (1:1,000), an antibody to σ_1_R (1:500), and an antibody to Na^+^-K^+^ ATPase (1:1,000). After rinsing in TBST buffer, the membranes were incubated with horseradish peroxidase-labeled secondary antibodies and developed using an enhanced chemiluminescence detection kit (Millipore, Billerica, MA, USA). Following visualization, the blots were stripped by incubation in stripping buffer (Restore; Pierce Biotechnology, Rockford, IL, USA) and then incubated with antibodies against PKC (1:1,000) or GAPDH (1:5,000). Western blot bands were then scanned and analyzed by Image J analysis software package (NIH). The optical density of specific bands was first normalized to the corresponding level of Na^+^-K^+^ ATPase or GAPDH and then normalized according to control levels.

### Behavioral investigations

Three different behavioral tests were carried out (09:00–14:00 h) under the following test sequence: open-field test (OFT) → forced swim test (FST) → tail suspension test (TST). The order of testing was chosen such that the test involving low-stress levels (OFT) took precedence over tests involving medium-stress levels (FST) and high-stress levels (TST; Di et al., 2017). The FST and OFT were performed at least 24 h apart while the TST and FST were performed at least 48 h apart, as previously reported (Di et al., 2017). These behavioral tests were captured by a video-monitor, and the data were analyzed using TopScan Lite 2.0 (Clever Sys., Reston, VA, USA).

Before the OFT, mice were moved to the testing area in their home cages and allowed to adapt to their new environment for at least 1 h. Each mouse was placed in a cuboid Plexiglas box (60 × 60 × 40 cm) with 15 lux lighting and allowed to explore freely for 5 min. The distance traveled within 5 min was measured to assess the state of movement (Dere et al., 2004).

The FST was performed as described previously (Zhang B. et al., 2017). In brief, swim sessions were conducted by placing mice in plastic cylinders (diameter: 12 cm; height: 24 cm) filled with water (23–25°C) to a height of 20 cm. The total duration of immobility during a 6-min test was then scored. A mouse was judged to be immobile when it stopped any movements except those that were necessary to keep its head above water.

The TST was carried out by taping the tail of a mouse onto a rod 60 cm above the floor, as described previously (Zhou et al., 2014).

#### Data analysis/statistics

**Figure 1:**
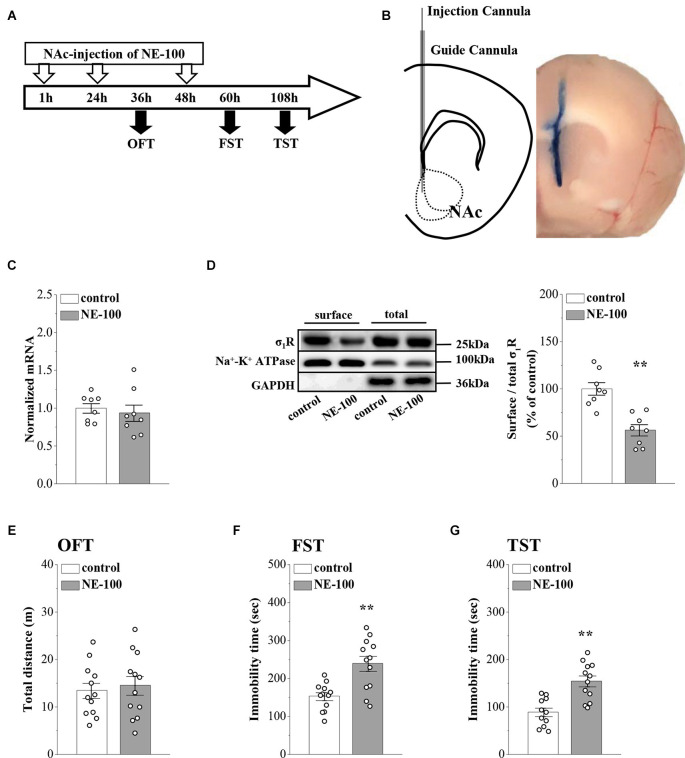
Repeated injection of NE-100 in the NAc induces depressive-like behaviors. **(A)** The time course of the experimental procedure. **(B)** Schematic diagram of the NAc (left panel) and image of the NAc injected with Evans-blue (right panel). **(C)** The level of *σ_1_R* mRNA in the NAc was examined at the end of the behavioral tests (*n* = 8 mice per group; Student’s *t*-test). **(D)** Representative Western blots of biotin-labeled surface proteins (surface) and total proteins (total) σ_1_R in the NAc. Na^+^-K^+^ ATPase served as internal control and GAPDH served as a negative control. Bar graphs indicate the ratio of proteins at the cell surface to their total levels. **p < 0.01 vs. control mice (*n* = 8 mice per group; Student’s *t*-test). **(E)** Bar graphs show the distance traveled in OFT in control mice and NE-100 mice (*n* = 12 mice per group; Student’s *t*-test). **(F,G)** Bar graphs show the immobility time of FST and TST in control mice and NE-100 mice. **p < 0.01 vs. control mice (*n* = 11 mice in control group of TST and *n* = 12 mice in other groups; Student’s *t*-test).

**Figure 2:**
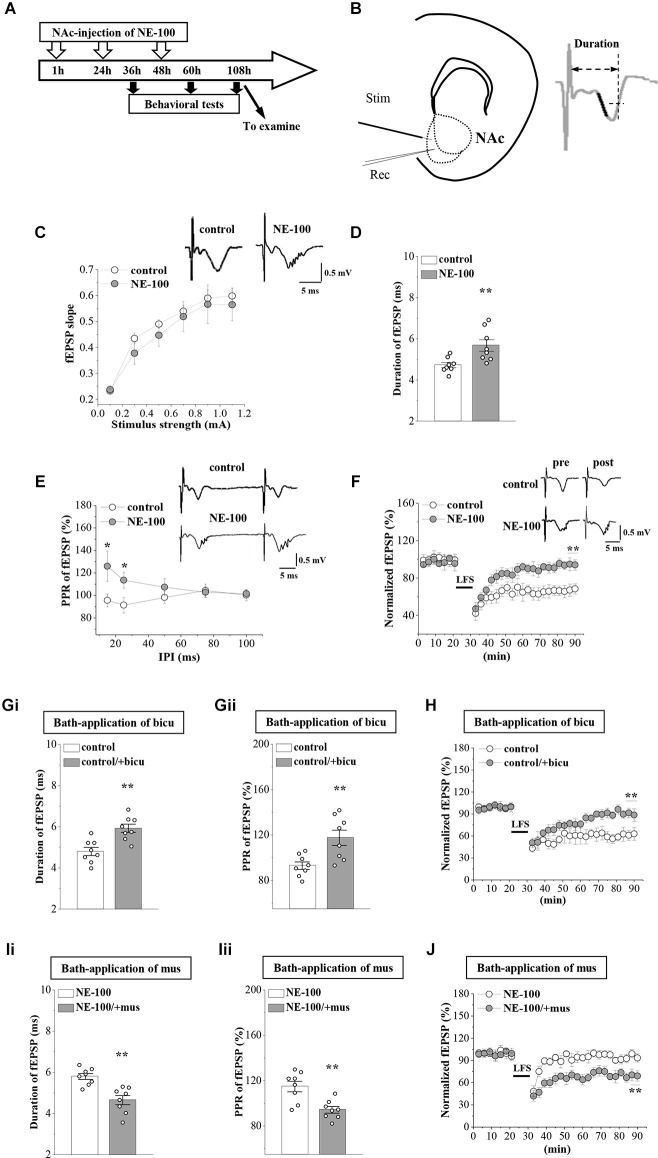
Repeated injection of NE-100 leads to synaptic dysfunction. **(A)** The timecourse of the experimental procedure. **(B)** Schematic illustrating sites of stimulating electrode (Stim) and recording electrode (Rec) in the shell of NAc (left panel). The right panel shows a typical trace. The dark mark of the initial descending phase indicates the 10% to 90% data points of the slope, and the two-way arrow indicates the duration. **(C)** Input-output (I-O) curve in the NAc. Each point represents the group mean value of fEPSP slopes against stimulus intensity from 0.1 mA to 1.1 mA in control mice and NE-100 mice. Representative traces of fEPSP evoked at 0.5 mA stimulus intensity in control mice and NE-100 mice (*n* = 8 slices/4 mice per group; repeated-measures ANOVA). **(D)** Bar graphs show the duration (ms) of fEPSP in control mice and NE-100 mice. **p < 0.01 vs. control mice (*n* = 8 slices/4 mice per group; Student’s-*t* test). **(E)** PPR (%) of fEPSP slopes was plotted against interpulse intervals (IPIs) ranging from 15 ms to 100 ms. Traces represent fEPSPs evoked by stimulation pulses delivered with a 25 ms IPI. *p < 0.05 vs. control mice (*n* = 8 slices/4 mice per group; repeated-measures ANOVA). **(F)** LTD induction by delivering low-frequency stimulation (LFS) in control mice and NE-100 mice. A solid line indicates when LFS was given. Traces show fEPSPs pre- and post-LFS. **p < 0.01 vs. control mice (*n* = 8 slices/4 mice per group; Mann-Whitney U test). **(Gi,Gii)** Bar graphs show the duration (ms) of fEPSP and the PPR value (25 ms IPI) in vehicle-treated and bicuculline (bicu)-treated control mice. **p < 0.01 vs. vehicle-treated mice (*n* = 8 slices/4 mice per group; Student’s *t*-test). **(H)** LTD induction by LFS in slices of control mice treated with bicuculline (bicu). **p < 0.01 vs. vehicle-treated mice (*n* = 8 slices/4 mice per group; Mann-Whitney U test). **(Ii,Iii)** Bar graphs show the duration (ms) of fEPSP and the PPR value (25 ms IPI) in vehicle-treated and muscimol (mus)-treated NE-100 mice. **p < 0.01 vs. vehicle-treated mice (*n* = 8 slices/4 mice per group; Student’s *t*-test). **(J)** LTD induction in slices of NE-100 mice treated with muscimol (mus). **p < 0.01 vs. vehicle-treated mice (*n* = 8 slices/4 mice per group; Mann-Whitney U test).

All outcome analyses were carried out by an independent investigator who was blinded to the treatment conditions and animal groupings. All data were retrieved and processed using the Microcal Origin 9.1 software program (Origin Lab, Northampton, MA, USA). All data were expressed as means ± standard error (SE) and were analyzed by SPSS software (SPSS, RRID: SCR-002865, version 18.0). All normally distributed data (determined by the Shapiro-Wilk test) were performed by the Grubb’s test to determine outliers (*p* < 0.01), and outliers in Figures 1G, 6B, and 6C were removed from the analysis. Differences between two groups were evaluated by the Student’s *t*-test (for normally distributed data). One-way analysis of variance (ANOVA) and two-way ANOVA were used to detect statistical significance between two or more groups on a single independent variable or two independent variables, respectively. With regards to the analysis of electrophysiological data: (1) input-output (I-O) function was assessed by measuring fEPSP slopes evoked by stimulating intensities from 0.1 to 1.1 mA. The duration of fEPSPs was measured as the time between the stimulus (measured at the midpoint of the stimulus artifact) to the time when the fEPSP amplitude decayed to half that of the maximal amplitude (Figure 2B; Yang et al., 2014); (2) the PPR was calculated with the following formula: (fEPSP_S2_/fEPSP_S1_) × 100, in which fEPSP_S1_ and fEPSP_S2_ represented the fEPSP slopes evoked by the first and second stimulations, respectively; and (3) for LTD analysis, the fEPSP slopes were normalized to the mean baseline slope over the last 10 min before LFS and plotted over time. The 20% lower values of the fEPSP slopes 50–60 min after delivering LFS than baseline was considered as maintenance of LTD. The effects of control and NE-100, along with the drugs applied in different groups, were determined by testing data from the last 10 min of recordings with the Mann-Whitney U test (as the data were not normally distributed). Repeated-measures ANOVA (with Greenhouse-Geisser corrections if necessary) was used to analyze I-O data and PPR. ANOVA, followed by Bonferroni’s *post-hoc* test, was performed when data showed homogeneity of variance. *P*-values < 0.05 were considered statistically different.

## Results

### Repeated injection of NE-100 into NAc leads to reduced surface expression of σ_1_r and depressive-like behaviors

In our previous study, we reported that σ_1_R gene knockout or the systematic administration of the σ_1_R inhibitor NE-100 induced or worsened depressive-like behaviors in mice (Sha et al., 2015; Zhang B. et al., 2017; Zhang S. et al., 2017). To test the specific effect of σ_1_R antagonist in the NAc on depressive-like behaviors in mice, we microinjected the NAc region of 12-week-old mice with NE-100 (0.15 nmol/mouse/day) for 3 days (NE-100 mice; Figures 1A,B). To clarify the effect of NE-100 administration on σ_1_R expression, we examined the level of σ_1_R in the NAc of control mice and NE-100 mice at the end of the behavioral tests. RT-PCR results showed that the administration of NE-100 did not affect the mRNA levels of σ_1_R in the NAc of mice (*t*_(14)_ = 0.511, *p* = 0.618; Figure 1C). The σ_1_R protein at approximately 25 kDa was observed in the NAc of control and NE-100 mice. In comparison with control mice, the total amount of σ_1_R protein was not significantly altered in NE-100 mice (*t*_(14)_ = −0.155, *p* = 0.879; Figure 1D), notably, the cell surface protein level of σ_1_R was significantly reduced in NE-100 mice (*t*_(14)_ = 4.939, *p* < 0.001), suggesting a downregulation of σ_1_R activity in the cell membrane.

We then examined spontaneous activity and depressive-like behaviors by the open-field test (OFT), forced swim test (FST), and tail suspension test (TST). As shown in Figure 1E, the total distance traveled in the OFT was not significantly different when compared between control mice and NE-100 mice (*t*_(22)_ = −0.419, *p* = 0.679). In comparison with control mice, the NE-100 mice exhibited prolongation of immobility time in the FST (*t*_(22)_ = −3.862, *p* < 0.001; Figure 1F) and TST (*t*_(21)_ = −4.444, *p* < 0.001; Figure 1G), thus indicating a state of despair. These results indicate that the impairment of σ_1_R in the NAc induces depressive-like behaviors in mice.

### Impaired synaptic function involved with GABA_A_R in NE-100 mice

Synaptic plasticity in the NAc is involved in depressive-like behavior caused by chronic unpredictable stress (Wang et al., 2010). To determine whether an impairment of σ_1_R function affects synaptic function in the NAc, we recorded field excitatory postsynaptic potentials (fEPSPs) by stimulating the NAc in brain slices obtained from control and NE-100 mice (Figure 2B). The mice were decapitated on day 2.5 after the last administration of NE-100 (at the end of the behavioral tests; Figure 2A) and brain slices were prepared to record field potentials. To evaluate the basal properties of the NAc, an I-O curve was generated by plotting fEPSP slopes against stimulation intensities from 0.1 mA to 1.1 mA. Repeated measures ANOVA found no significant interactions for the control mice and NE-100 mice over six different stimulation intensities on the fEPSP slope (*F*_(5,35)_ = 0.430, *p* = 0.824; Figure 2C). Interestingly, when compared with the single fEPSP waveform in control mice, the same stimulation elicited a multi-spike fEPSP waveform in the NAc of NE-100 mice (upper right panel in Figure 2C). The duration of fEPSP in NE-100 mice was significantly longer than that in control mice (*t*_(14)_ = −3.094, *p* = 0.008; Figure 2D). In addition, the paired-pulse ratio (PPR) in NE-100 mice was significantly larger than those in control mice with an interpulse interval (IPI) of 15–25 ms (15 ms IPI: *p* = 0.025; 25 ms IPI: *p* = 0.045; Figure 2E); values with the different IPIs (50–100 ms) showed no change (*p* > 0.05). Notably, the fEPSP slopes reduced by approximately 35% following the delivery of low-frequency stimulation (LFS) over 60 min in control slices, indicating LTD maintenance. The same LFS protocol did not induce a stable reduction of the fEPSP slopes in brain slices from NE-100 mice (*U*_(8,8)_ = 0, *p* < 0.001; Figure 2F).

According to our previous studies, the increased PPR indicates dysfunctional GABA_A_R-mediated inhibition (Zhang T. et al., 2017). To test the involvement of GABA_A_R in synaptic dysfunction in the NAc region of NE-100 mice, the NAc slices were treated with the GABA_A_R antagonist bicuculine (10 mM) or agonist muscimol (10 mM) for 30 min. The application of bicuculine caused an increase in the duration of fEPSP (*p* = 0.001; Figure 2Gi) and PPR value (IPI: 25 ms; *p* = 0.006; Figure 2Gii) in control mice. In addition, the application of muscimol led to the decrease in the duration of fEPSP (*p* = 0.001; Figure 2Ii) and PPR (*p* = 0.002; Figure 2Iii) in NE-100 mice. Furthermore, the application of bicuculline caused LTD to be unsustainable in control mice (*p* < 0.001; Figure 2H), while the application of muscimol recovered LTD in NE-100 mice (*p* < 0.001; Figure 2J). Therefore, these findings indicate that an impairment of σ_1_R function in the NAc attenuates synaptic plasticity and GABA_A_R-mediated inhibition.

### Reduced GABA_A_R and D2R expression in NE-100 mice

**Figure 3:**
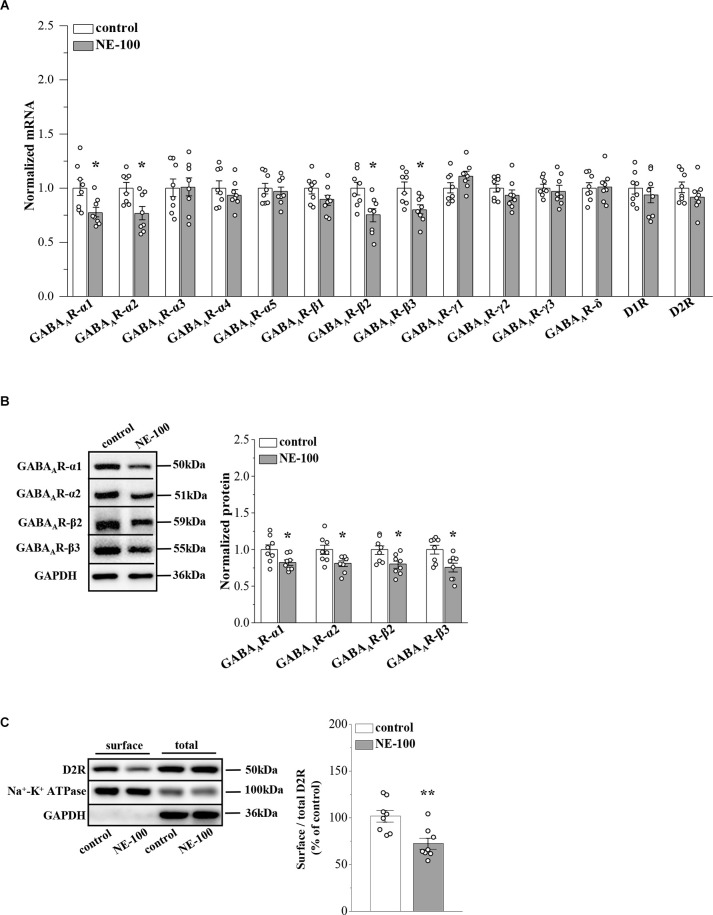
Repeated injection of NE-100 suppresses GABA_A_R and D2R expression. **(A)** The levels of *GABA_A_R-α1-5*, *-β1-3*, *-γ1-3*, *-δ* subunits, and *D1R*, *D2R* mRNA in the NAc were examined at the end of the behavioral tests. *p < 0.05 vs. control mice (*n* = 8 mice per group; two-way ANOVA). **(B**) Bar graphs show levels of GABA_A_R-α1, GABA_A_R-α2, GABA_A_R-β2, and GABA_A_R-β3 protein in the NAc normalized by the level of GAPDH were normalized by control levels. *p < 0.05 vs. control mice (*n* = 8 mice per group; two-way ANOVA). **(C)** Representative Western blots of biotin-labeled surface proteins (surface) and total proteins (total) D2R in the NAc. Na^+^-K^+^ ATPase served as internal control and GAPDH served as a negative control. Bar graphs indicate the ratio of proteins at the cell surface to their total levels. **p < 0.01 vs. control mice (*n* = 8 mice per group; Student’s *t* -test).

GABA_A_R-α2, -α4, -δ, and other α and β subunits were reported to be expressed at levels ranging from strong to weak in the NAc region of male mice (Hortnagl et al., 2013). To investigate the mechanisms underlying the abnormal synaptic properties and plasticity in the NAc of NE-100 mice, we investigated the expression of GABA_A_R and D1 and D2 receptors (D1R and D2R) in the NAc by RT-PCR and Western blotting, respectively, on day 2.5 after the last administration of NE-100 (timeline see Figure 2A). For mRNA levels, the results of a two-way ANOVA revealed a significant main effect of repeated NE-100 treatment (*F*_(1,196)_ = 18.235, *p* < 0.001; Figure 3A). The main effect of genes (*F*_(13,196)_ = 1.721, *p* > 0.05) or NE-100 treatment × genes interaction was not significant (*F*_(13,196)_ = 1.721, *p* > 0.05). Bonferroni *post-hoc* analyses revealed that the levels of *GABA_A_R-α1* (*p* = 0.048), *GABA_A_R-α2* (*p* = 0.017), *GABA_A_R-β2* (*p* = 0.010), and* GABA_A_R-β3* (*p* = 0.014) mRNA in NE-100 mice were significantly reduced when compared to those in control mice. There were no significant differences between control mice and NE-100 mice with respect to the other *GABA_A_R* subunits or *D1R* and *D2R* mRNA (*p* > 0.05). Furthermore, the results of a two-way ANOVA revealed a significant main effect of repeated NE-100 treatment on the protein levels of GABA_A_R subunits (*F*_(1,56)_ = 27.563, *p* < 0.001; Figure 3B). The main effect of the GABA_A_R subunits (*F*_(3,56)_ = 0.138, *p* > 0.05)as well as the interaction (*F*_(3,56)_ = 0.138, *p* > 0.05)were not significant. Bonferroni *post-hoc* analyses revealed that the levels of GABA_A_R-α1 (*p* = 0.031), GABA_A_R-α2 (*p* = 0.017), GABA_A_R-β2 (*p* = 0.011) and GABA_A_R-β3 (*p* = 0.030) protein in NE-100 mice were significantly lower than those in control mice.

σ_1_R can be complex with D2R on the cell membrane (Borroto-Escuela et al., 2020). To analyze whether inhibition of σ_1_R in the NAc affects D2R internalization, we investigated the distribution of D2R on the membrane surface by using a cell surface biotinylation approach. The level of D2R at the cell surface in the NAc of NE-100 mice was significantly reduced when compared to controls (*t*_(14)_ = 3.448, *p* = 0.004; Figure 3C). Quantification of the total amount of D2Rs in the NAc revealed no difference between the NE-100 mice and control mice (*p* > 0.05).

### Reduced D2R-mediated PKC activity suppresses GABA_A_R expression in NE-100 mice

**Figure 4:**
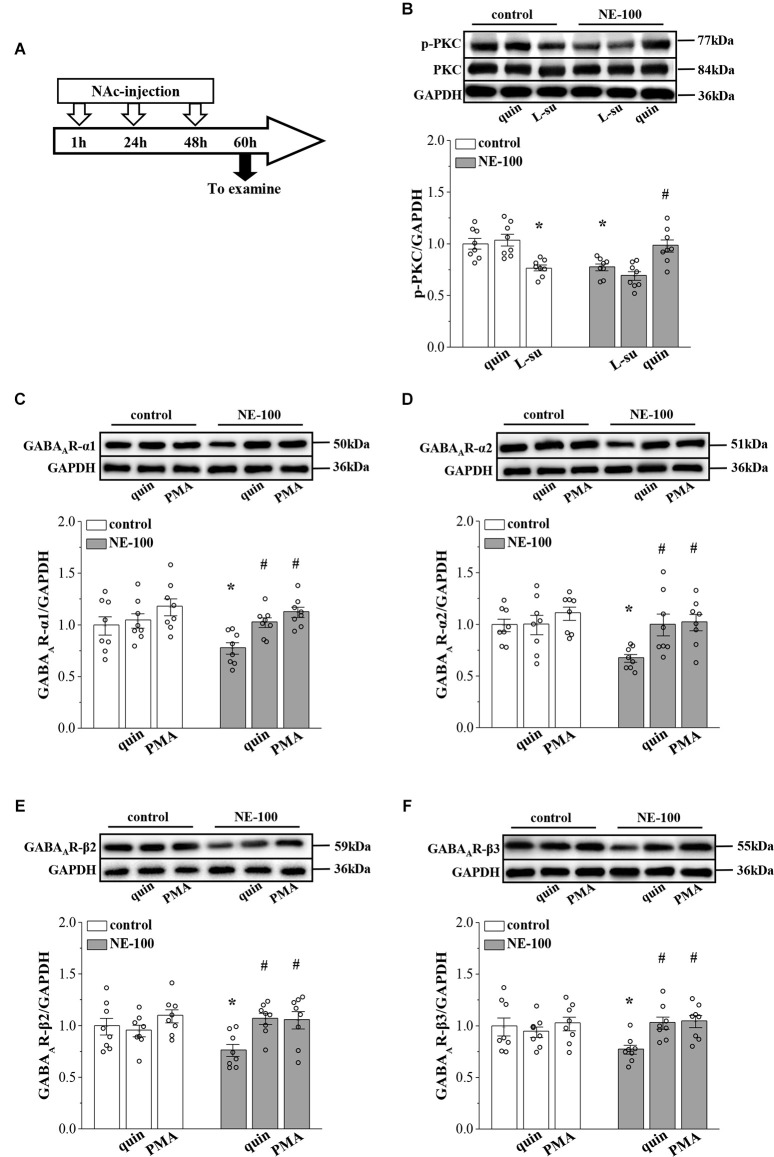
Repeated injection of NE-100 reduces PKC signaling leading to decreased GABA_A_R expression. **(A**) Time chart of the experimental procedure. **(B**) Representative blots of phospho-PKC in the NAc of control mice treated with quinpirole (quin) or L-sulpiride (L-su), and NE-100 mice treated with L-sulpiride (L-su) or quinpirole (quin). Densitometric values of phospho-PKC normalized by the PKC protein were normalized by control levels. *p < 0.05 vs. control mice; ^#^*p* < 0.05 vs. NE-100 mice (*n* = 8 mice per group; two-way ANOVA). **(C**–**F**) Bar graphs show levels of GABA_A_R-α1, GABA_A_R-α2, GABA_A_R-β2, and GABA_A_R-β3 protein in the NAc of control mice and NE-100 mice treated with NAc-injection of quinpirole (quin) or PMA. Densitometric values normalized by the level of GAPDH were normalized by control levels. *p < 0.05 vs. control mice; ^#^*p* < 0.05 vs. vehicle-treated NE-100 mice (*n* = 8 mice per group; two-way ANOVA).

In our previous studies, we showed that the inhibition of D2R indirectly suppresses the GABAergic inhibitory circuit by affecting the activity of PKC (Zhang T. et al., 2017). Here, we further examined the levels of PKC phosphorylation (phospho-PKC) within the NAc (Figure 4A). For the level of phospho-PKC, the results of a two-way ANOVA revealed significant main effects of repeated NE-100 treatment (*F*_(1,42)_ = 8.934, *p* = 0.005; Figure 4B) and drug treatment (*F*_(2,42)_ = 18.099, *p* < 0.001), but the interaction was not significant (*F*_(2,42)_ = 2.013, *p* > 0.05). Bonferroni *post-hoc* analyses revealed that the level of phospho-PKC was significantly reduced in NE-100 mice than in control mice (*p* = 0.025); this was rescued by injecting the NAc with quinpirole, a D2R agonist (*p* = 0.045), yet quinpirole had no effect on the level of phospho-PKC in control mice (*p* > 0.05). Administration of the D2R antagonist L-sulpiride significantly reduced the levels of phospho-PKC in control mice (*p* = 0.014), but not in NE-100 mice (*p* > 0.05). There was no significant difference in the levels of PKC proteins when compared between control mice and NE-100 mice (*p* > 0.05).

Next, we microinjected the NAc region of control and NE-100 mice with D2R agonist quinpirole or PKC activator PMA (Figure 4A) to examine the effects on the level of GABA_A_R subunits. The results of a two-way ANOVA revealed significant main effects of drug treatment on the protein level of GABA_A_R subunits (α1: *F*_(2,42)_ = 7.762, *p* < 0.001; Figure 4C; α2: *F*_(2,42)_ = 4.752, *p* = 0.014; Figure 4D; β2: *F*_(2,42)_ = 4.411, *p* = 0.018; Figure 4E; β3: *F*_(2,42)_ = 3.502, *p* = 0.041; Figure 4F), but not the interaction (α1: *F*_(2,42)_ = 1.286, *p* > 0.05; α2: *F*_(2,42)_ = 2.320, *p* > 0.05; β2: *F*_(2,42)_ = 2.325, *p* > 0.05; β3: *F*_(2,42)_ = 3.050, *p* > 0.05). Bonferroni *post-hoc* analyses revealed that in NE-100 mice, injecting the NAc with quinpirole or the PKC activator PMA rescued the protein levels of GABA_A_R-α1 (quinpirole: *p* = 0.037; PMA: *p* = 0.010), along with the -α2 (quinpirole: *p* = 0.014; PMA: *p* = 0.011), -β2 (quinpirole: *p* = 0.010; PMA: *p* = 0.011), and -β3 (quinpirole: *p* = 0.017; PMA: *p* = 0.011) subunits. In contrast, neither quinpirole nor PMA had a significant effect on the level of GABA_A_R subunits in the NAc of the control mice (*p* > 0.05).

### Mechanisms involved in the synaptic dysfunction in NE-100 mice

**Figure 5:**
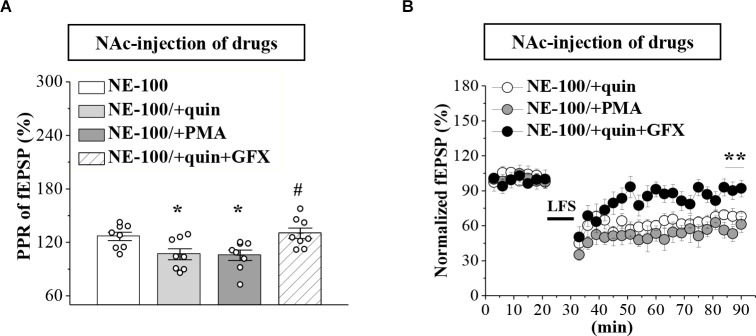
Repeated injection of NE-100 impairs GABA_A_R-regulated LTD through reduced PKC activity. **(A**) Bar graphs show the PPR (25 ms IPI) in the slices of NE-100 mice treated with NAc-injection of quinpirole (quin), PMA, or the co-administration of quinpirole and GF109203X (quin+GFX). *p < 0.05 vs. vehicle-treated mice; ^#^*p* < 0.05 vs. quin-treated mice (*n* = 8 slices/4 mice per group; one-way ANOVA). **(B**) LTD induction in NE-100 mice treated with NAc-injection of quinpirole (quin), PMA, or the co-administration of quinpirole and GF109203X (quin+GFX). **p < 0.01 vs. quin-treated mice (*n* = 8 slices/4 mice per group; Mann-Whitney U test).

To test the involvement of D2R-mediated PKC activity in synaptic dysfunction caused by reduced GABA_A_R in the NAc, the NE-100 mice were treated with NAc-injection of quinpirole, PMA, or coadministered quinpirole and GF109203X for 3 days (for a time chart of the experimental procedure, see Figure 4A). The results of a one-way ANOVA revealed a significant main effect of drug treatment (*F*_(3,28)_ = 5.724, *p* = 0.003; Figure 5A). Bonferroni *post-hoc* analyses revealed that the application of quinpirole or PMA led to a significant reduction in the PPR (*p* < 0.05). In parallel, quinpirole or PMA resulted in an approximately 35% reduction in the fEPSP slopes over 60 min post-LFS (Figure 5B). In addition, the effects of quinpirole on the PPR (*p* < 0.05) or LTD (*p* < 0.01) were sensitive to the co-injection of GF109203X.

### Impaired LTD is involved in depressive-like behaviors of NE-100 mice

**Figure 6:**
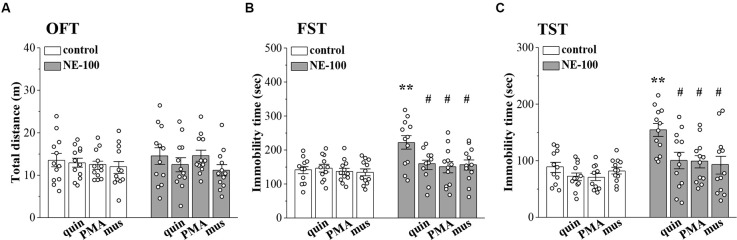
Involvement of impaired LTD in the NAc in depressive-like behaviors. **(A**–**C)** Bar graphs show the distance traveled in OFT, and the immobility time in FST and in TST of control mice, NE-100 mice, and mice treated with NAc-injection of quinpirole (quin), PMA, or muscimol (mus). **p < 0.01 vs. control mice; ^#^*p* < 0.05 vs. vehicle-treated NE-100 mice (*n* = 11 mice of quin-treated group in FST, *n* = 11 mice of vehicle-treated control group and PMA-treated control group in TST, and *n* = 12 mice in the other groups; two-way ANOVA).

To investigate the relationship between altered synaptic function and depressive-like behaviors, we conducted OFT, FST, and TST evaluations on days 2–4 after the administration of NE-100 and drugs (for a time chart of the experimental procedure, see Figure 1A). For OFT (Figure 6A), the results of a two-way ANOVA revealed no significant main effect of repeated NE-100 treatment (*F*_(1,88)_ = 0.216, *p* > 0.05), drug treatment (*F*_(3,88)_ = 1.105, *p* > 0.05), or the interaction (*F*_(3,88)_ = 0.429, *p* > 0.05). Bonferroni *post-hoc* analyses revealed that the distance traveled in the OFT did not differ significantly between control and NE-100 mice and of the drugs applied groups (*p* > 0.05). For FST (Figure 6B) and TST (Figure 6C), the main effect of repeated NE-100 treatment (FST: *F*_(1,86)_ = 12.89, *p* = 0.001; TST: *F*_(1,86)_ = 19.433, *p* < 0.001), drug treatment (FST: *F*_(3,86)_ = 3.974, *p* = 0.011; TST: *F*_(3,86)_ = 5.391, *p* = 0.002) and the interaction (FST: *F*_(3,86)_ = 3.206, *p* = 0.027; TST: *F*_(3,86)_ = 3.014, *p* = 0.047) were significant. Bonferroni *post-hoc* analyses revealed that injecting the NAc with quinpirole (FST:*p* = 0.034; TST: *p* = 0.015), PMA (FST: *p* = 0.010; TST: *p* = 0.011), and muscimol (FST: *p* = 0.012; TST: *p* = 0.010) rescued the prolongation of immobility time in the FSTand TSTin NE-100 mice. Nevertheless, neither quinpirole, PMA, nor muscimol had a significant effect on the immobility time in FST (*p* > 0.05) and TST (*p* > 0.05) in control mice.

## Discussion

In this study, we investigated the influence of σ_1_R antagonist NE-100 in the NAc on synaptic plasticity and depressive-like behaviors. Microinjection of NE-100 into the NAc for 3 days did not affect the total amount of σ_1_R, whereas it caused a decrease in σ_1_R surface expression, suggesting downregulation of its activity in the cell membrane. As a result, our findings provide the first *in vivo* evidence that inhibition of σ_1_R in the NAc impairs LTD by reducing GABA_A_R expression, leading to depressive-like behaviors. Besides, there is a difference when compared to previous results: we reported that the GABA-activated current remained unaltered in the hippocampal dentate gyrus (DG) of σ_1_R knockout mice, while the NMDA-activated current was reduced (Sha et al., 2013). Our present results showed that repeated inhibition of σ_1_R in the NAc caused a reduction in the expression of GABA_A_R subunits. In addition, an NMDAR agonist did not rescue LTD in NE-100 mice (Supplementary Figure S1B). Possible explanations for these conflicting data are that the cellular properties of the NAc differ from those of immature cells in the DG, and that there may be key differences between experiments involving gene knockouts and pharmacological inhibitors. Further studies on the effects of downregulated σ_1_R on NMDAR, GABA_A_R, and GABA_B_R in the NAc MSNs are in progress.

A principal finding in the present study is that the membrane levels of D2R were reduced in the NAc when σ_1_R was deficient. The activity of σ_1_R is closely related to the function of dopamine receptors. It has been found that in cultured cells, and in animal striatum, σ_1_R can complex with D2R to form a heterodimer, thus maintaining its stability and acting as a target for D2R ligands to activate or inhibit downstream signaling pathways (Borroto-Escuela et al., 2019, 2020). Our results showed that repeated inhibition of σ_1_R in the NAc did not affect the transcriptional and total protein levels of D2R; however, membrane protein expression was significantly reduced, thus suggesting that downregulated σ_1_R in the NAc causes a reduction in D2R-mediated downstream signaling. The activation of σ_1_R prevents intracellular calcium dysregulation and increases intracellular calcium responses (Katnik et al., 2006; Choi et al., 2022). Intracellular Ca^2+^ signals can affect the activity of neuronal calcium sensor-1 (NCS-1); NCS-1 inhibits the internalization and desensitization of D2R in a Ca^2+^-dependent manner (Kabbani et al., 2002). Therefore, we hypothesize that σ_1_R complexes with D2R to form heterodimers that stabilize the membrane by anchoring D2R and that downregulated σ_1_R causes an imbalance in the distribution of D2R in cells, thus resulting in the reduced expression of D2R in the membrane. Another explanation is that repeated inhibition of σ_1_R indirectly causes the abnormal distribution of D2R in cells by reducing intracellular Ca^2+^ signaling, thus resulting in a reduction in the endogenous ligand binding sites and overall functionality.

A previous study reported that σ_1_R agonists induce the activation of PKC (Morin-Surun et al., 1999). In the present study, we found that the levels of phospho-PKC in the NAc of NE-100 mice were lower than those in control mice. The D2R agonist could recover the level of phospho-PKC in NE-100 mice, without affecting the activation of PKC in control mice; the D2R antagonist caused the decline of phospho-PKC in control mice, but not in NE-100 mice. Moreover, neither agonist nor antagonist of D1R, have these effects (Supplementary Figures S2Ai and Aii), suggesting that σ_1_R regulates PKC activity by affecting D2R. Hong et al. (2016) reported that agonists of D2R enhanced PKC signaling. In our previous study, we demonstrated that D2R downregulation in BLA caused a reduction in PKC phosphorylation levels (Zhang T. et al., 2017). D2R activates the downstream phospholipase C (PLC)-diacylglycerol (DAG)-PKC signaling pathway *via* Gαi proteins (Yao et al., 2008). Thus, inhibition of σ_1_R in the NAc leads to reduced levels of PKC phosphorylation *via* the downregulation of D2R.

Electrophysiological results showed that inhibition of σ_1_R in the NAc caused multi-peak-like change, a prolonged duration in the fEPSP, and an increased PPR; collectively, these results indicated a weakening in the functionality of the GABA_A_R-mediated inhibitory circuit in amygdale (Delaney and Sah, 2001; Zhang T. et al., 2017). In line with our results showing that inhibition of GABA_A_R resulted in an increased PPR value and extended fEPSP duration in the NAc of control mice, and activation of GABA_A_R corrected the increased PPR and extended fEPSP duration in the NAc of NE-100 mice. The expression levels of GABA_A_R-α1, along with the -α2, -β2, and -β3 subunits were all decreased in NE-100 mice. Previous immunocytochemical studies revealed that the rodent brain the NAc expresses high levels of GABA_A_R-α1, along with the -α2, -β2, and -β3 subunits, with MSN dendrites expressing α2 and β3 subunits and interneurons expressing α1 and β2 subunits (Schwarzer et al., 2001; Boyes and Bolam, 2007). Thus, the reduced expression of these subunits causes the functionality of the GABA_A_R inhibitory circuit to be diminished. It was previously demonstrated that activation of D2R promotes the embedding of GABA_A_R in protrusions and the formation of new inhibitory synapses (Li et al., 2011). D2R knockout mice exhibit attenuated GABAergic neurotransmission (An et al., 2004); the long-term activation of D2R has been shown to increase postsynaptic GABA_A_R cluster density in striatal MSNs (Lalchandani et al., 2013). The present results showed that D2R activation increased the expression levels of GABA_A_R subunits and restored the PPR in the NAc of NE-100 mice; these effects were blocked by PKC inhibition (Supplementary Figures S3Aii–Dii). Activation of the PKC increased the expression of GABA_A_R subunits and restored PPR of the NAc in NE-100 mice. In contrast, no obvious effect of either activation of D2R or PKC on the GABA_A_R subunits in the NAc of control mice was found. The inhibition of D2R and PKC reduced the expression of GABA_A_R in control mice (Supplementary Figures S3Ai–Di), thus suggesting that D2R exerts a regulatory effect on GABA_A_R *via* PKC signaling. Activated PKC is known to contribute to GABA_A_R function, transport, and cell surface stability (Field et al., 2021). The activation of PKC promotes phosphorylation of α and β subunits to increase the surface expression of GABA_A_R (Luscher et al., 2011; Nakamura et al., 2015). Consistent with the present results, our previous study confirmed that the inhibition of PKC reduces the expression levels of GABA_A_R subunits, which consequently leads to a functional downregulation of GABA_A_R in BLA (Zhang T. et al., 2017). Collectively, these results indicate thatinhibition of σ_1_R in the NAc causes the reduction of GABA_A_R subunits expression *via* downregulation of D2R-mediated PKC signaling, which further leads to a downgrade in GABA_A_R function.

Basal synaptic transmission in the NAc was not affected in NE-100 mice. However, the maintenance of LTD was impaired in the NAc of NE-100 mice. The activation of GABA_A_R restored LTD maintenance in NE-100 mice while the inhibition of GABA_A_R resulted in impaired LTD in control mice. A previous study showed that D2R inhibition affects LTP in the BLA by influencing presynaptic CB1R activation; however, the current results suggest that CB1R activation did not affect LTD in the NAc of NE-100 mice (Supplementary Figure S1C). The activation of D2R and GABAR facilitates the inwards flow of Ca^2+^ mediated by voltage-gated calcium channels (Guatteo et al., 2004). Consistent with this observation, the administration of the GABA_A_R agonist muscimol in the NAc brain slices in adolescent mice was found to depolarize neuronal membrane projection and promote the maintenance of LTD (Zhang et al., 2016). Thus, repeated inhibition of σ_1_R in the NAc impairs LTD by reducing GABAergic function. GABAergic inhibition participates in regulating depressive-like states: male α2 subunit knockout mice appear depressive-like behavior (Vollenweider et al., 2011); in male mice, α2-containing GABA_A_Rs on D2R-positive but not on D1R-positive neurons promote resiliency to chronic social defeat stress (Benham et al., 2021). Additionally, similar to the effects of GABA_A_R agonists, the activation of D2R or PKC restored LTD maintenance in the NAc while also correcting depressive-like behaviors in NE-100 mice; and yet these agonists had no significant effect on the behaviors of control mice. These results suggest that the LTD of synaptic plasticity in the NAc is closely related to depression. Impaired LTD in the NAc is known to contribute to depressive-like behaviors induced by chronic unpredictable stress in mice (Wang et al., 2010). In a mouse model of chronic mild stress, excessive activation of glycogen synthase kinase-3β led to decreased synaptic plasticity in the NAc; this subsequently led to reduced adaptive flexibility to stress and induced depressive disorders (Aceto et al., 2020). A reduction in the GABAergic synapses of the NAc was previously correlated with depressive-like behaviors and stress susceptibility (Heshmati et al., 2020).

## Conclusions

The results from the present study indicate that inhibition of σ_1_R in the NAc led to the suppression of GABA_A_R expression through a reduction in D2R/PKC activation, thus impairing LTD and leading to depressive-like behaviors. The present study provides a new insight into the mechanisms underlying depressive disorders and identifies potential therapeutic targets.

## Data Availability Statement

The original contributions presented in the study are included in the article/Supplementary material, further inquiries can be directed to the corresponding author/s.

## Ethics Statement

The animal study was reviewed and approved by Animal Research Institute and Ethical Committee of Nanjing Medical University.

## Author Contributions

SS conceived and designed the experiments. YQ and WX performed the field potential recording, Western blotting, and all statistical analysis. KL and QL undertook the RT-PCR analysis. XC and YW carried out the animal care and behavioral examination. LC and SS drafted the manuscript. All authors contributed to the article and approved the submitted version.

## Funding

This study was supported by the National Natural Science Foundation of China (31600835).
