# Supplementary material for: Repeated inhibition of sigma-1 receptor suppresses GABAA receptor expression and long-term depression in the nucleus accumbens leading to depressive-like behaviors
Source: Front Mol Neurosci. 2022 Sep 30;15:959224. doi: 10.3389/fnmol.2022.959224 (PMC9563353; doi:10.3389/fnmol.2022.959224)
Supplement: Supplementary file 1 [file Data_Sheet_1.docx]

Supplementary Material

## Figure S1


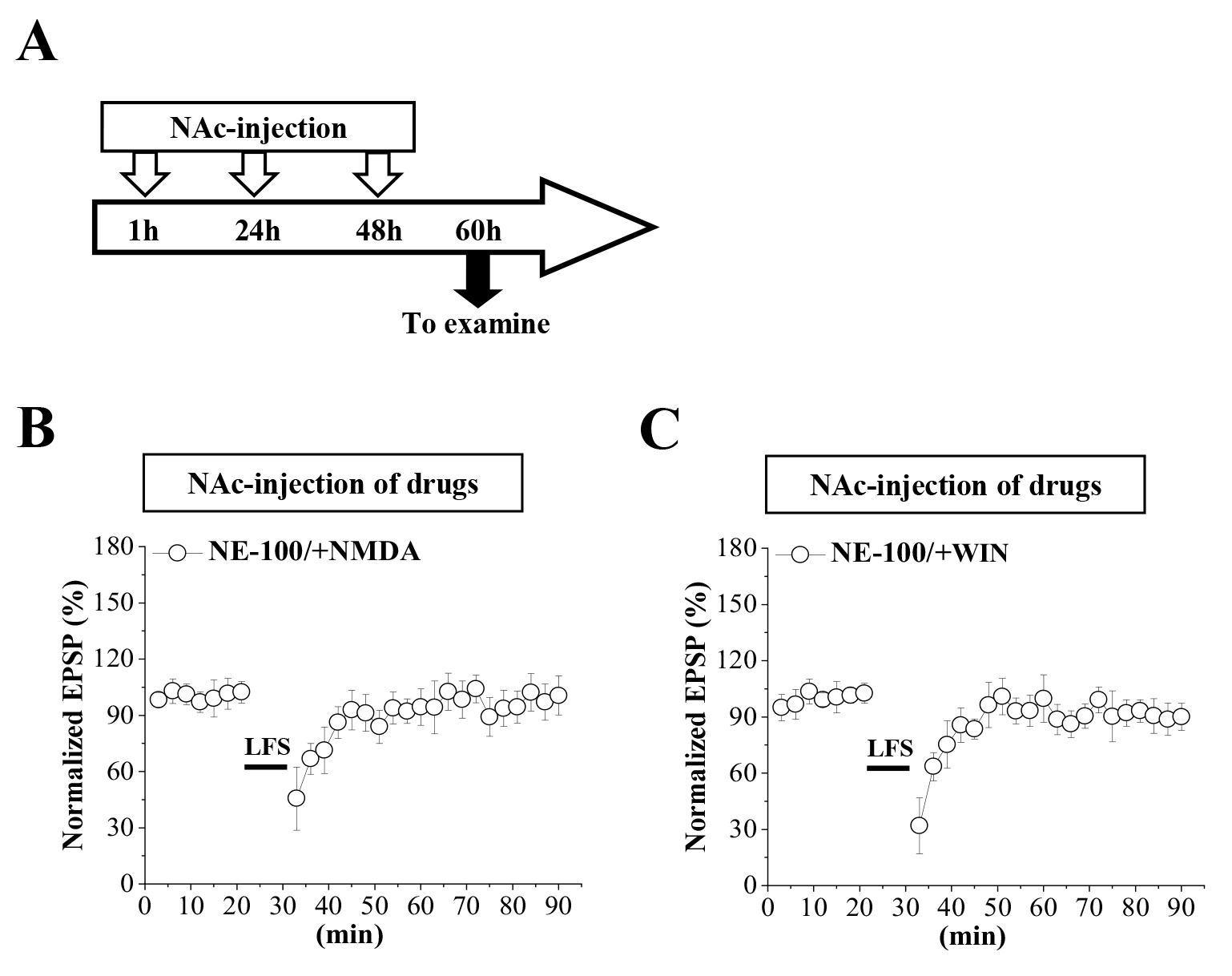


**Supplementary Figure 1.** Injecting the NAc of NE-100 mice with NMDA receptor (NMDAR) agonist NMDA or CB1 receptor agonist WIN55,212-2 (WIN; time chart of experimental procedure in **Figure S1A**) did not rescue LTD maintenance in the NAc (**B** and **C**).

## Figure S2


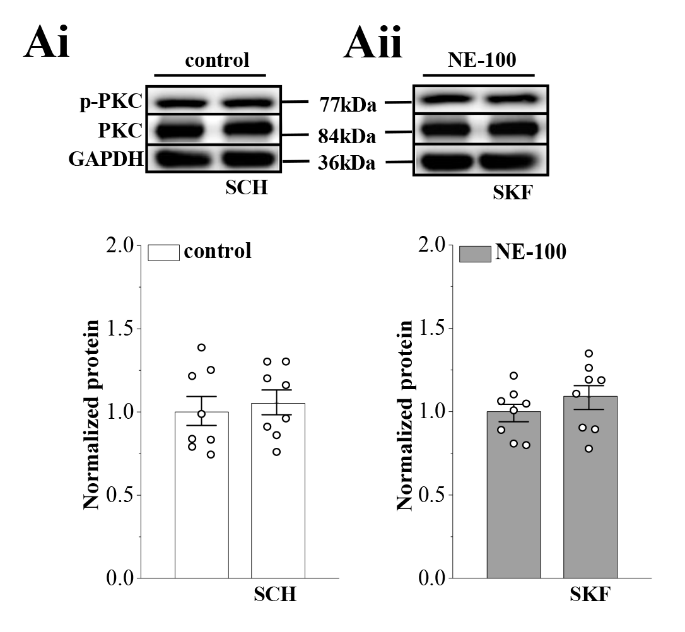


**Supplementary Figure 2.** The administration of NAc-microinjecting of D1R antagonist SCH23390 (SCH) in control mice, or NAc-microinjecting of D1R agonist SKF38393 (SKF) in NE-100 mice (time chart of experimental procedure in **Figure S1A**) did not affect PKC activity (*p* > 0.05; **Figure S2Ai** and **Aii**).

## Figure S3


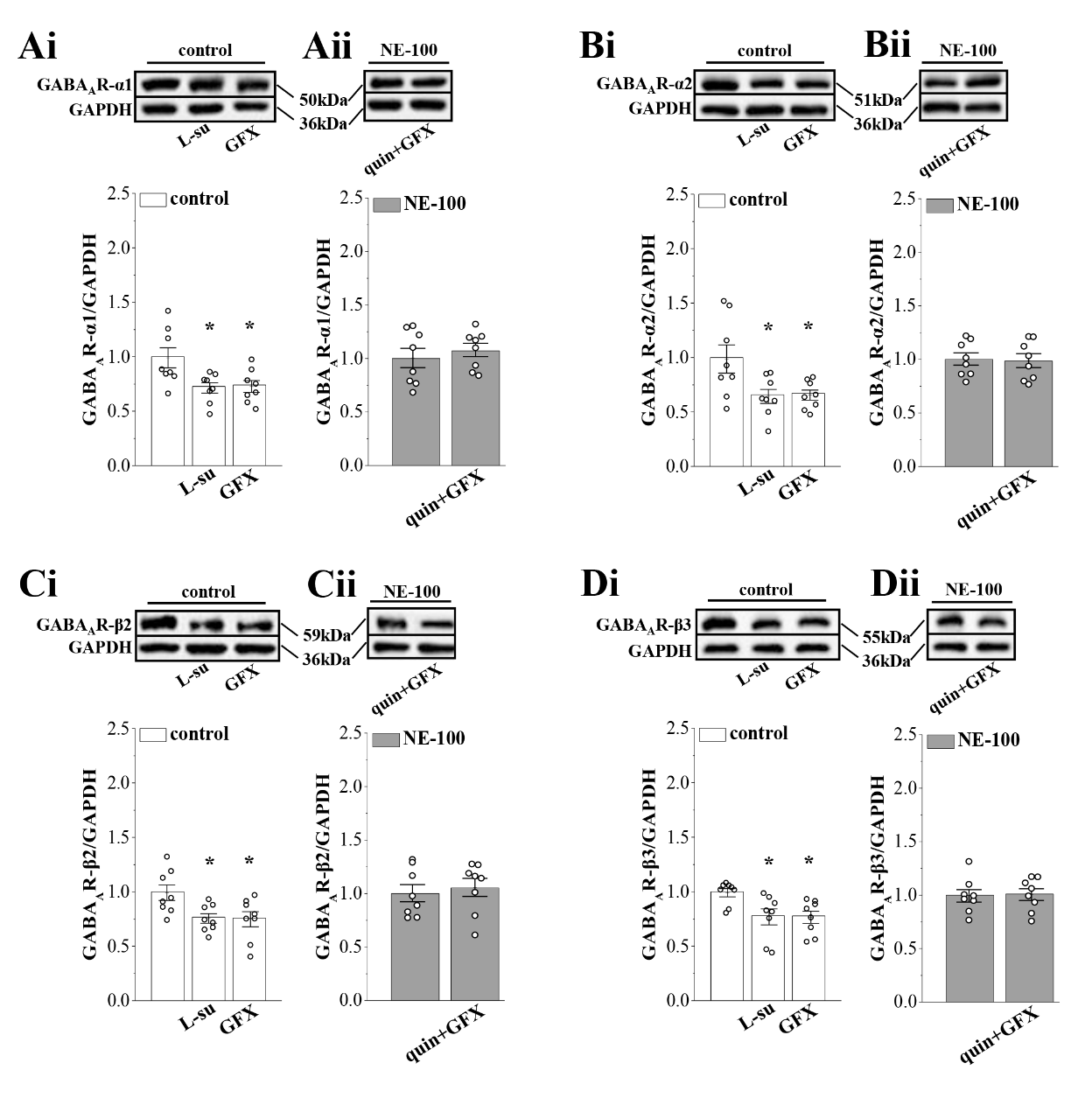


**Supplementary Figure 3.** In control mice, injecting the NAc with L-sulpiride (L-su) or the PKC inhibitor GF109203X (GFX) led to a significant reduction in the protein levels of GABA_A_R-α1 (L-sulpiride: *p* = 0.026; GF109203X: *p* = 0.036; **Figure S3Ai**), along with the -α2 (L-sulpiride: *p* = 0.034; GF109203X: *p* = 0.044; **Figure S3Bi**), -β2 (L-sulpiride: *p* = 0.043; GF109203X : *p* = 0.037; **Figure S3Ci**) and -β3 subunits (L-sulpiride: *p* = 0.037; GF109203X: *p* = 0.033; **Figure S3Di**). Injecting the NAc with coadministered of quinpirole (quin) and GF109203X showed no significant effect on GABA_A_R expression in NE-100 mice (*p* > 0.05; in **Figure S3Aii**–**Dii**).
